# Coupling of Oxy- and Deoxyhemoglobin concentrations with EEG rhythms during motor task

**DOI:** 10.1038/s41598-017-15770-2

**Published:** 2017-11-13

**Authors:** Piotr Lachert, Dariusz Janusek, Przemyslaw Pulawski, Adam Liebert, Daniel Milej, Katarzyna J. Blinowska

**Affiliations:** 1 Nalecz Institute of Biocybernetics and Biomedical Engineering Polish Academy of Science, Trojdena 4, Warsaw, 02-109 Poland; 2 Department of Biomedical Physics, Faculty of Physics, University of Warsaw, Pasteura 5, 02-093 Warszawa, Poland

## Abstract

A relationship between the brain rhythmic activity and the hemodynamic response was studied using the simultaneous measurement of electroencephalogram (EEG) and the functional near-infrared spectroscopy (fNIRS) during a motor task (self-paced right finger movements) for 10 subjects. An EEG recording with a 32-electrode (10-10) system was made and the hemodynamic response was obtained using 8 optodes placed over the sensorimotor cortex on both hemispheres. During the task an increase in oxyhemoglobine (HbO) was accompanied by a decrease in deoxyhemoglobine (HbR) concentration and a decrease in amplitudes (desynchronisation) of alpha (8–13 Hz) and beta (13–30 Hz) EEG rhythms. These phenomena were prominent in the hemisphere contralateral to the moving finger. The delays between the hemodynamic and electrophysiological variables were on average 2.8 s. Highly significant (p < 0.0001) negative Pearson correlations were found between HbO and alpha (r^2^ = −0.69) and HbO and beta (r^2^ = −0.54) rhythms. Positive correlations r^2^ = 0.5 between these rhythms and HbR were found.

## Introduction

Nowadays a large bulk of evidence concerning topographical and functional aspects of neural activity comes from the neuroimaging methods such as fMRI and fNIRS. However, the fundamental relationship between cerebral changes of hemodynamics and brain activity is hardly known. Therefore, integrating electrophysiological and hemodynamic signals (fNIRS, BOLD) and understanding of the neurovascular coupling is in the center of attention of the scientific community.

Information about neurovascular coupling may be derived from multimodal measurements of hemodynamic and electric activity of the brain using fMRI or fNIRS combined with EEG or Local Field Potentials. There is a number of technical and methodological problems connected with simultaneous fMRI/EEG recording. In particular, EEG data registered during fMRI acquisition is disturbed by artifacts induced by magnetic fields gradients. Blood oxygenation level dependent (BOLD) signal measured with fMRI is primarily sensitive to the venous blood flow, whereas NIRS detects the hemodynamic changes at the capillary level as well^1^. BOLD reflects the content of deoxyhemoglobin in blood, since in a strong magnetic field the presence of paramagnetic (a high spin state) deoxyhemoglobin in red blood cells makes their magnetic susceptibility different from the diamagnetic plasma. NIRS measures both oxygenated (HbO) and deoxygenated (HbR) hemoglobin concentration changes in the superficial layers of the human cortex. While the concentration of HbO is expected to rise after the activation of cortex due to the higher blood flow, HbR is washed out and its concentration decreases^2^. The factors which make NIRS potentially advantageous for assessment of neurovascular coupling are: i) both deoxyhemoglobin and oxyhemoglobin changes may be measured, and ii) there is evidence that neuronal function is supported to the large degree by brain capillary oxygenation, which is detected by NIRS.

The relationship between hemodynamic responses and spectral characteristics of neural activity is hardly known and divergent. While hemodynamic changes are related to the number of activated neurons, the EEG rhythm amplitudes depend on the number of neurons acting synchronously. The rhythmic neural activity plays an important role in information processing in the brain providing coupling mechanisms and synchronisation between neural populations. It is, therefore, of great importance to find the relationship between oscillatory EEG activity and hemodynamic variables. The problem of the relationship between EEG rhythms and the BOLD signal was reviewed e.g. in ref.^3^.

There is less evidence concerning the relationship between fNIRS and EEG than between BOLD signal and EEG, moreover, the results seem to be divergent. Simultaneous fNIRS and EEG recordings showed an increase in HbO and HbR during epileptic seizures; an initial decrease in HbR was followed by its increase^4^, which may be explained by the fact that an increase in oxygen metabolism is not sufficiently compensated during the seizure. Obrig *et al*.^5^ found that an increase in the amplitude of the early components of visual evoked potential induced by a checkerboard stimulus was accompanied by an increase in HbO and a decrease in HbR concentration. The results of median nerve stimulation^6^ revealed an increase in HbO concentration at the contralateral primary sensorimotor cortex which then spread to the more posterior and ipsilateral regions. The changes in HbO were stronger with an increase in the frequency of electrical stimulation. Herrmann *et al*.^7^ investigated brain responses to negative and positive stimuli. The EEG showed an increase in early posterior negativity over the occipital cortex for both positive and negative stimuli (in comparison to neutral ones) which was accompanied by a decrease in HbR. However, the results for HbO were divergent.

A simultaneous recording of EEG/fNIRS was performed with the aim to enhance the performance of the hybrid Brain Computer Interface^8^. The incorporation of HbO concentration into the set of parameters improved the classification accuracy of motor imagery performance by 5% on average in comparison to the EEG based classifier. HbO yielded higher accuracy levels than HbR for motor imagery. Interestingly, in the imagery condition HbO decreased in both hemispheres.

The intracranial recording of brain activity combined with fNIRS did not supply clear evidence concerning the neurovascular coupling mechanisms. Simultaneous epidural fNIRS and cortical electrophysiological measurements^9^ showed significant correlations between underlying neural activity modulation and hemodynamic peak time occurrence (positive for HbO, negative for HbR), but not for peak amplitude.

A heuristic model concerning BOLD/EEG relationship was elaborated by Kilner *et al*.^10^. According to the developed model, an increase in the BOLD signal for higher frequency bands is accompanied by its decrease for lower frequencies. Indeed, some simultaneous fMRI/EEG measurements confirmed this hypothesis. The multimodal EEG/fMRI studies indicated that alpha rhythm is negatively correlated with the BOLD signal e.g.: Goldman *et al*.^11^, Martinez-Montez. *et al*.^12^. The reduction of BOLD was found also in the experiment concerning low-frequency EEG entrainment^13^. In the higher frequency bands: 17–23 Hz and 24–30 Hz the authors reported predominantly positive correlations between EEG and fMRI. However, Leopold *et al*.^14^ reported that very slow EEG activity fluctuations in the monkey visual cortex were connected with the increase in the BOLD signal.

The correlation between the EEG rhythms and fNIRS response investigated by Koch *et al*.^15^ who considered the dependence between the individual alpha frequency (IAF) peak and hemodynamic responses. The authors reported that high IAF correlates with a low oxygenation response and low IAF with a higher oxygenation level. They conjectured that the relationship between IAF, neuronal and vascular response depended on the size of recruited neuronal population.

The aim of our study was to investigate the relationship between EEG rhythms and HbO and HbR concentrations using simultaneous measurement of EEG and fNIRS. Brain rhythms play a crucial role in synchronisation of neural populations and information transfer. Finding the correlation between the changes in blood oxygenation levels and changes in the amplitudes of EEG rhythms has a potential to elucidate the relationship between hemodynamic and electrophysiological processes in the brain. The experiment which elicits very characteristic spectral features in the frequency bands above 8 Hz is the motor task which is accompanied by the so-called event related synchronisation (ERD) and desynchronisation (ERS) phenomena occurring in the motor cortex^16^. The paradigm of the present study involves fNIRS and EEG measurements during a finger movement task. The measurements of synchronisation and desynchronisation of EEG rhythms together with fNIRS made it possible to find a relationship between the phenomena directly connected with the synchronized activity of neural populations and hemodynamic processes.

## Results

**Figure 1:**
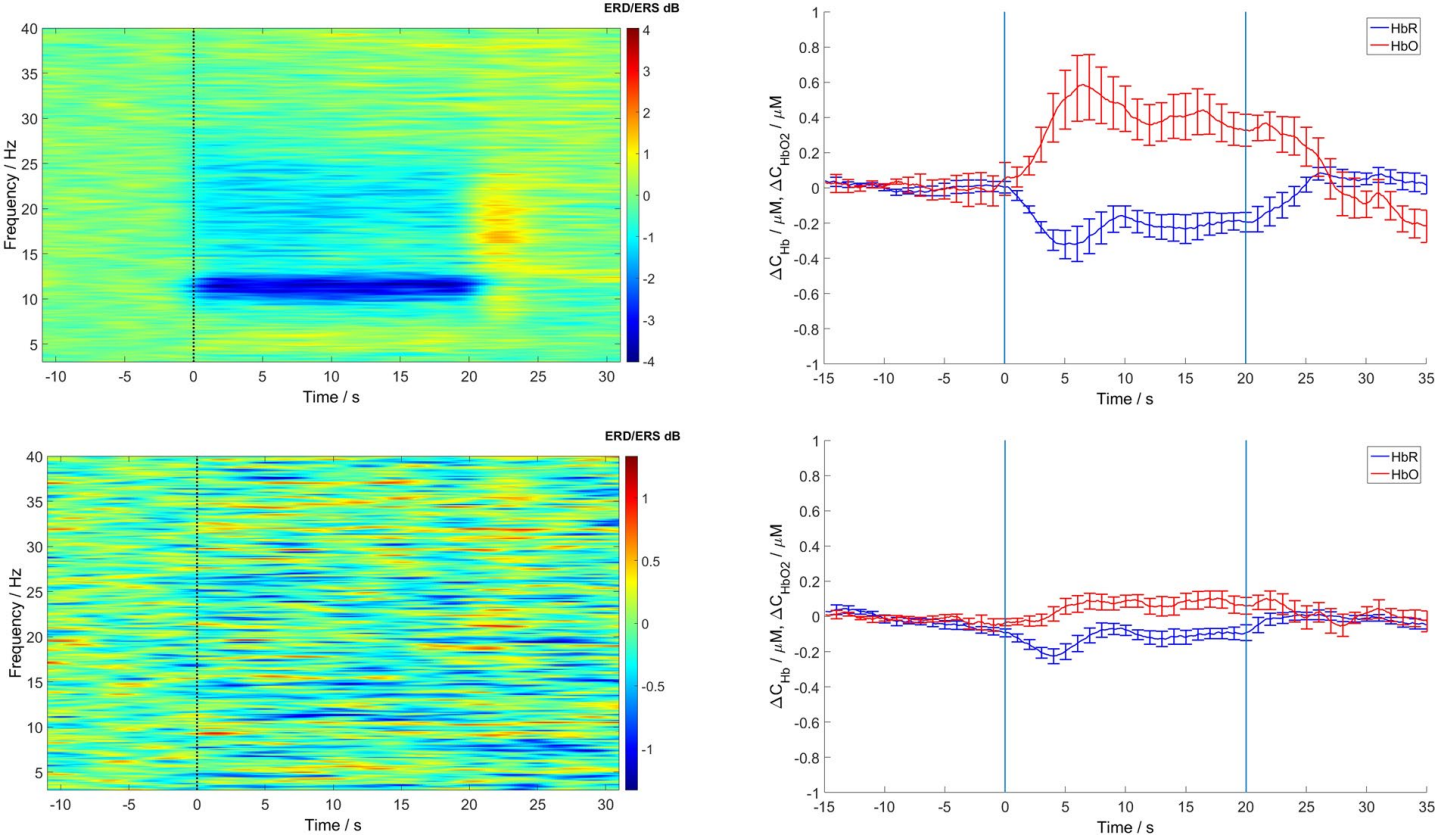
ERD/ERS distributions (left panels) and concentration changes in time for HbO (red) and HbR (blue) - (right panels) averaged over subjects. Upper pictures correspond to the most reactive electrode - C3 and corresponding optode; lower pictures to the electrode located in the right hemisphere - Cp2 and corresponding optode. Movement period from 0 to 20 s. At the very left of ERD/ERS panels - spectra.

In Fig. 1 ERD/ERS time courses for two electrodes: C3, Cp2 and time evolutions of HbO and HbR for corresponding optodes, averaged over subjects, are displayed.

Hand movements are controlled mainly by the central areas of the hemisphere contralateral to the moving hand. The C3 electrode located above the motor cortex of the left hemisphere shows pronounced changes in the time-frequency distribution of EEG which are accompanied by an increase in HbO concentration in the corresponding optode number 1; Cp2 located in the right hemisphere does not show changes in the movement period, also, concentration changes of HbO and HbR in the right hemisphere were much less pronounced. An increase in HbO and a decrease in HbR during the movement accompanied by a decrease in the amplitude (desynchronisation) in the alpha and beta bands was observed for all subjects. The maximal changes of HbO and HbR were visible in the left hemisphere mostly at the optodes which corresponded to the C3 and C1 electrodes located above the motor cortex of the right finger. These changes were accompanied by desynchronisation of alpha and beta rhythms at the corresponding electrodes. In our experiment the HbO and HbR curves for the right hemisphere (ipsilateral with respect to the moving finger) usually had low amplitudes. The hemodynamic responses lasted longer than the effective movement period, namely around 25 s, depending on the subject.

In the movement period an increase in HbO and a decrease in HbR were observed in all the subjects except one person in whom an increase in HbO was accompanied by an increase in HbR. An application of the Kolmogorow-Smirnow test showed the significance of changes in HbO and HbR concentration during movement with respect to the rest period at the level of p < 0.0001 except for two subjects in whom the changes in HbR concentration were insignificant. The HbR data on those subjects were excluded from further analysis.

**Figure 2:**
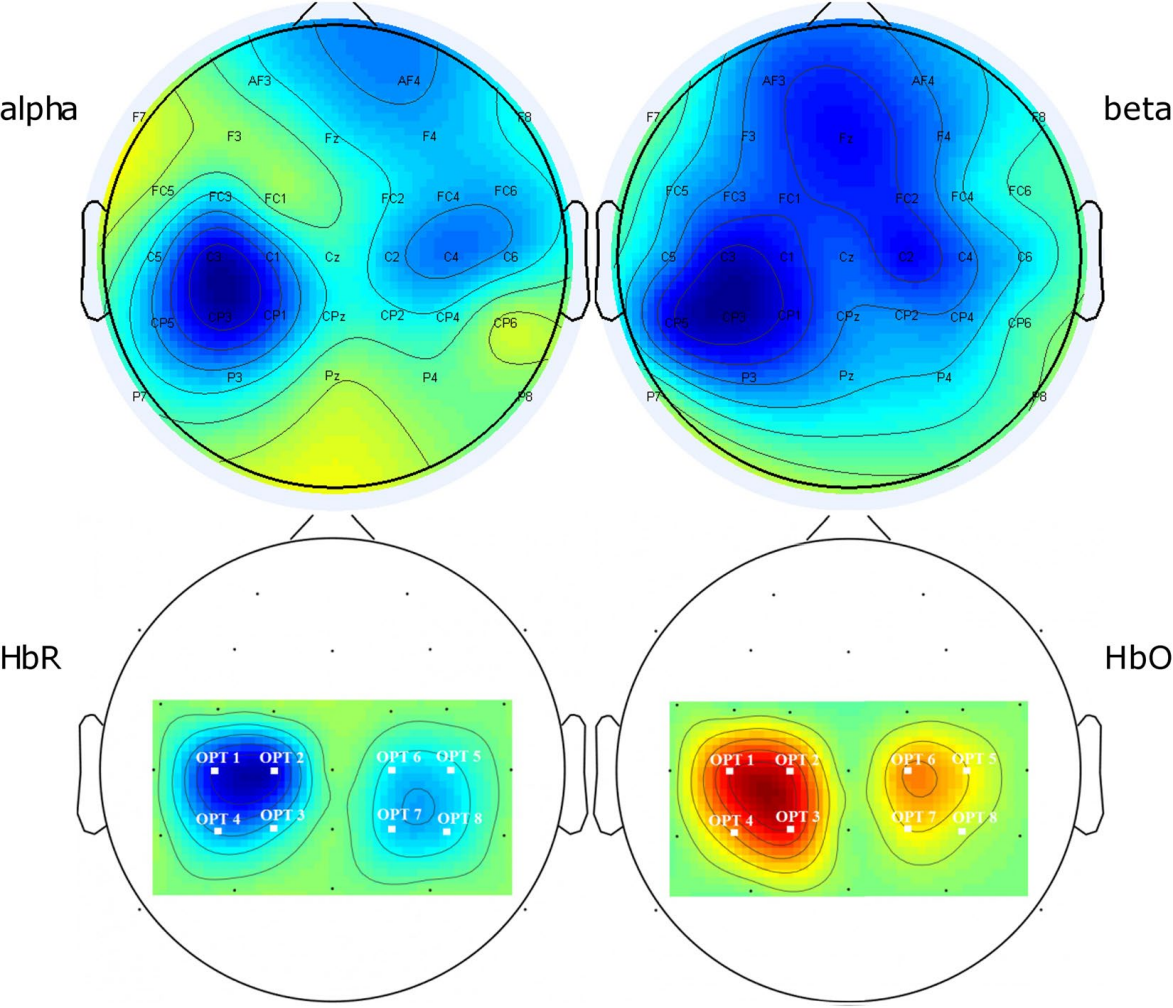
Topographic distribution of desynchronisation in alpha and beta bands (upper pictures) and a decrease in HbR and an increase in HbO (lower pictures) quantified by their z-scores. Blue colors illustrate a decrease whereas red and yellow colors represent an increase.

The maps illustrating topographically ERD/ERS responses in alpha and beta frequency bands and HbO/HbR concentration changes during movement averaged over subjects are shown in Fig. 2.

The pattern of an increase in HbO corresponds very well with a decrease in HbR. The lateralization of both responses, although not complete, is easy to see. The comparison of the HbO and HbR maps with the desynchronisation maps have only illustrative value and can be only qualitative as ERS/ERD maps were constructed for 32 electrodes and HbO/HbR maps were evaluated for only 8 optodes. One can observe that the lateralization (prevalence of changes in the hemisphere contralateral to the moving finger) well visible for hemodynamic changes also occurs for alpha and beta rhythms.

**Figure 3:**
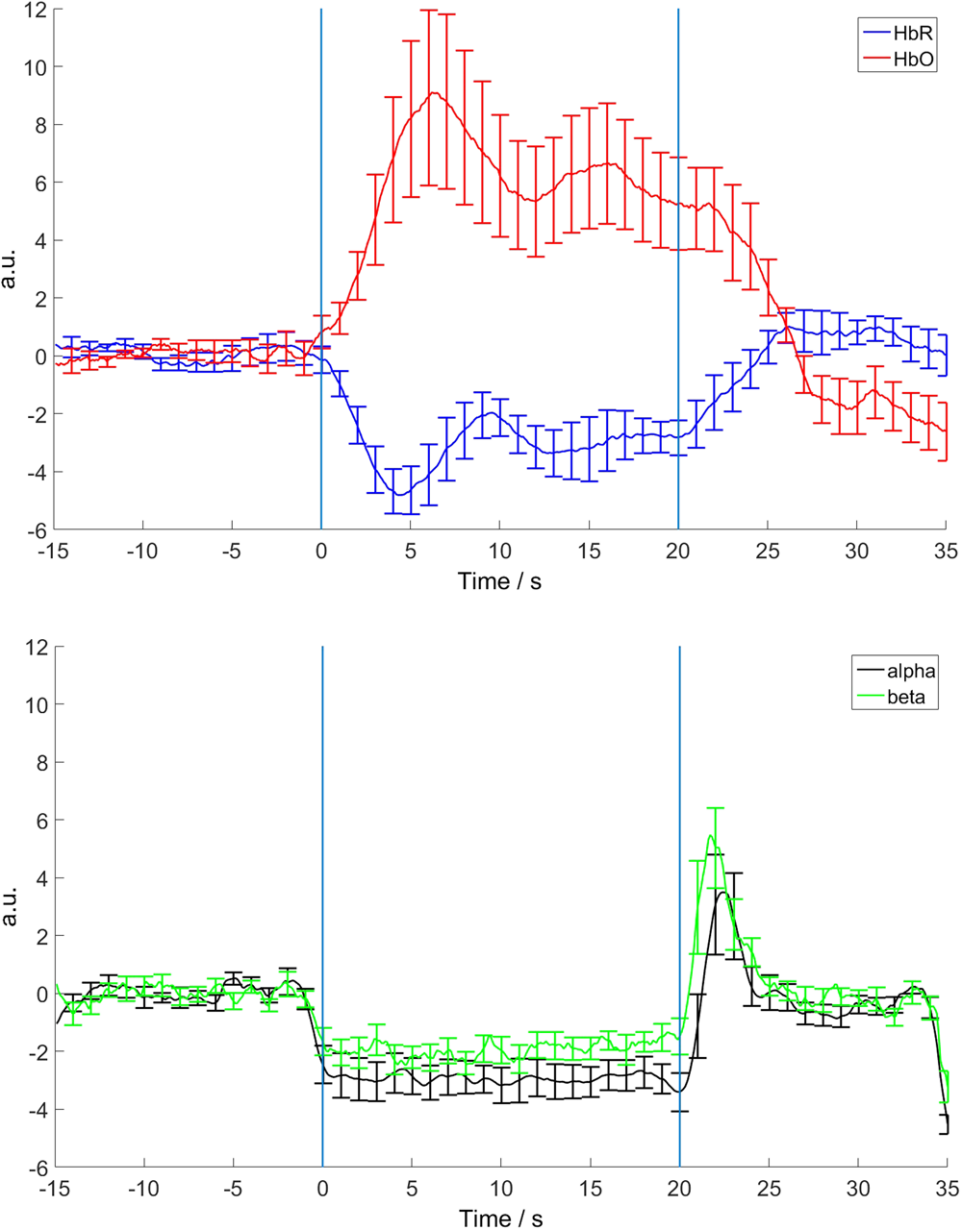
The time evolutions of HbO concentration (red), HbR concentration (blue) (upper picture), alpha ERD/ERS (black), beta ERD/ERS (green) (lower picture) for the most reactive electrode (C3) and the corresponding optode averaged over subjects. Movement period from 0 to 20 s.

The time evolutions of HbO, HbR and ERD for alpha and beta rhythms for the most reactive electrode (C3) and the corresponding optode averaged over subjects are shown in Fig. 3

It is easy to observe that a pronounced increase in HbO is accompanied by a decrease in HbR and decreases in alpha and beta rhythms amplitudes. The onsets of ERD for alpha and beta precede the start of the movement and at the end of the activation period a rapid resynchronisation of EEG rhythms occurs. The hemodynamic concentration changes are delayed with respect to movement start and continue for around 5 seconds after the end of movement.

The onsets of HbO, HbR, alpha and beta ERD and the onset differences between the hemodynamic and electrophysiological variables averaged over subjects are shown in Table 1. (Low pass filtering of the fNIRS signals at 0.4 Hz limited the range of calculated delays to the values above 2.0 s, however, smaller delays could hardly be expected).

**Table 1:** Latencies of the onset of HbO, HbR, alpha and beta signals, differences between onsets and correlations between hemodynamic and electrophysiological signals.

Latencies of the onsets of signals with respect to the start of the movement in seconds			
HbO	HbR	alpha	beta
2.71 ± 1.29	2.67 ± 1.01	−0.16 ± 0.73	−0.03 ± 0.53
**Differences between onsets of respective signals in seconds**			
HbO - alpha	HbO – beta	HbR – alpha	HbR - beta
2.87 ± 2.02	2.74 ± 1.82	2.79 ± 1.80	2.67 ± 1.60
**Person correlations - r** ^**2**^ **between hemodynamic and electrophysiological signals**.			
HbO - alpha	HbO – beta	HbR – alpha	HbR - beta
−0.69 ± 0.16	−0.54 ± 0.32	0.59 ± 0.29	0.49 ± 0.22

In calculating the averages of the HbR onsets, the data on subjects showing statistically insignificant values of HbR changes were discarded and the two outliers (values differing from the mean by a factor of 2 or more) were eliminated. The values of onsets were calculated with respect to the start of movement; in cases of alpha and beta the values slightly preceded the movement since the brain electrical activity triggers the action. The hemodynamic variables were delayed in relation to electrophysiological ones on average by 2.8 s. The delay values with the errors are given in Table 1.

In order to quantitatively estimate the dependence between brain rhythms and hemodynamic response we calculated the Pearson correlation coefficient between the amplitude evolution of alpha and beta rhythms and the HbO/HbR levels for each subject. The correlations between hemodynamic and electrophysiological signals averaged over subjects are shown in the lower row of Table 1. For the relationship HbO – alpha, the negative correlation was the highest: r^2^ = −0.69 and the significance for all the subjects was p < 0.0001. The correlation for HbO - beta was also at the p < 0.0001 level except for one subject in whom p < 0.05. That subject had a very weak beta rhythm. For HbR-alpha we discarded the correlations for two subjects in whom the difference between HbR concentration changes during movement with respect to the rest period was not significant. In one of the remaining 8 subjects the significance was p < 0.03, in all the other subjects it was p < 0.0001. The average HbR-beta correlation was computed for 7 subjects. Two subjects were excluded due to the fact that the change in HbR concentration during movement with respect to the rest period was not significant. One subject was excluded because of a weak beta rhythm.

## Discussion

The present study showed high and significant correlations between brain rhythm amplitudes during the motor task and the HbO and HbR concentrations. While the HbO concentration correlated negatively with the desynchronisation of alpha and beta rhythms, the HbR concentration correlated positively with the decrease in the amplitudes of both rhythms. Alpha and beta rhythms occurring in the central parts of the head (Rolandic rhythms) are manifestation of synchronous activity of neural populations; the contribution of synchronously acting neurons to the EEG amplitude is proportional to their number N, whereas for non-synchronous populations it is proportional to $\sqrt{N}$ ^17^. Brain activation accompanying motor or sensory tasks reflected in hemodynamic changes does not only involve synchronously acting neural populations. The intrinsic electrical activity of brain consists of local field potentials (synchronous and asynchronous) and spikes (action potentials); their coupling with hemodynamic changes manifested by BOLD were studied e.g. in^18^ and revealed a highly complex and not fully understood relationship. NIRS measurements, which unlike BOLD enable possible detection of HbO/HbR concentration change, have potential to significantly contribute to understanding of neurovascular coupling.

Usually brain activation during particular tasks is connected with an increase in HbO as was the case for evoked responses e.g. Obrig *et al*.^5^, Takeuchi *et al*.^6^. Zama and Shimada^19^ investigated the relationship between readiness potential (RP) – a slow negative deflection occurring about 1000 ms before movement onset and HbO concentration. The RP potential was accompanied by a rise in HbO, the Pearson correlation between HbO and RP was not very high but significant: r^2^ = 0.235 (p = 0.03). The interpretation of this finding was that the NIRS signal reflects the process of electrical excitation. The coupling between HbO and the evolution of alpha and beta rhythms found by us had a higher level of significance and was much stronger albeit negative.

The coupling between the hemodynamic response and brain activity during a motor task was studied by Mackert *et al*.^20^ and Sander *et al*.^21^. They recorded simultaneously dc-magnetoencephalography (dcMEG) and time-resolved near-infrared spectroscopy (trNIRS) in the task involving finger movements. The bandwidth of the dcMEG signal (0.01 - 0.2 H) was similar to the one of the vascular response. This very low frequency MEG signal, which presumably reflected sustained preparatory activity of the neural populations to process the input, was accompanied by an increase in HbO concentration and a decrease in HbR signal. The onsets of HbO/HbR activation found in Mackert *et al*.^20^, namely: 5.4 ± 1.2 s (HbO and 4.8 ± 0.5 s (HbR) were longer than the ones found by us, however in the above reference the responses were calculated with respect to the time of cue occurrence, whereas in our case it was the start of the movement. The delay in the HbO concentration increase found by us (2.71 ± 1.29) matches within the error limits the one found by Wriessenagger *et al*.^22^ who reported for the right hand movement HbO delay (1.96 ± 1.16 s).

The relationship between alpha and beta rhythms with fMRI-BOLD in Primary Somatosensory and Motor Cortex was studied during the bimanual motor task by Ritter *et al*.^23^. The results showed an inverse correlation between electrophysiological rhythm strengths and the BOLD signal. The BOLD effect is primarily driven by the change in local deoxyhemoglobin concentration which depends on the combined changes in cerebral blood flow (CBF), cerebral metabolic rate of oxygen (CMRO2) and the cerebral blood volume (CBV)^24^. The changes of these variables have a conflicting effect on the BOLD response. Considering the complex mechanisms behind the generation of the BOLD signal, we may conclude that the findings of Ritter *et al*.^23^ are consistent with our results.

In the paper concerning a temporal comparison of BOLD and NIRS hemodynamic responses during a finger tapping task^25^ similarly to our work, an increase in HbO and a decrase in HbR were observed. As expected, the correlation between HbR and BOLD was stronger than the correlation between HbO and BOLD. The peak responses showed a temporal lag of BOLD versus a few-second delay of HbO, however, substantial inter-individual differences were present.

The problem of the lateralization of brain activation during a motor task is significant for e.g. BCI design. The literature concerning the lateralization effect present in motor tasks as assessed with fNIRS is divergent. The results of Mackert *et al*.^20^ did not reveal specific topographic features. Similarly, according to Wriessenagger *et al*.^22^ the HbO changes appeared bilaterally. On the other hand though, Obrig *et al*.^26^ and Horovitz and Gore *et al*.^27^ found clear lateralization during hand movement execution tasks.

In our study, in the alpha band the contralateral desynchronisation was stronger than in the ipsilateral one, where it extended to the frontal regions (Fig. 2). The lateralization effect was to some degree visible in HbR and most evident in HbO. The incomplete lateralization can be explained by the fact that the topographic features of brain activation in a motor task evolve with time. According to the literature, the ERD phenomenon is most prominent over the contralateral sensorimotor areas during motor preparation and extends bilaterally with movement initiation^28^, so it is most pronounced in short, cue-driven experiments. In case of prolonged self-paced movements usually applied in NIRS studies, the lateralization effect can be to a certain degree weakened. As we observed, the lateralization is also quite variable across the subjects. These factors: task protocol and inter-subject variability may influence the topography of brain reaction to stimulus and lead to discrepancies in the results. Finally, we can conjecture that for the protocol tailored to emphasize the preparatory phase of a motor task, the lateralization effect should occur, bringing promise to BCI studies.

Our results represent convincing evidence for the correlation between brain rhythmic activity and hemodynamic changes and contribute to the understanding of the neurovascular coupling phenomena.

## Methods

### Subjects

Sixteen subjects took part in the experiment. The subjects were treated in strict compliance with the Declaration of Helsinki. All the subjects were informed about the experimental procedures and gave a written consent. The experiment was approved by the Ethical Commission of the Warsaw Medical University. Six persons were eliminated from the further evaluation because of their too weak or too noisy fNIRS signal. The analysis was performed on the signals obtained from 10 subjects (the mean age was 28.1 years, ranging from 24 to 38 years, 5 females, 5 males).

### Procedure

**Figure 4:**
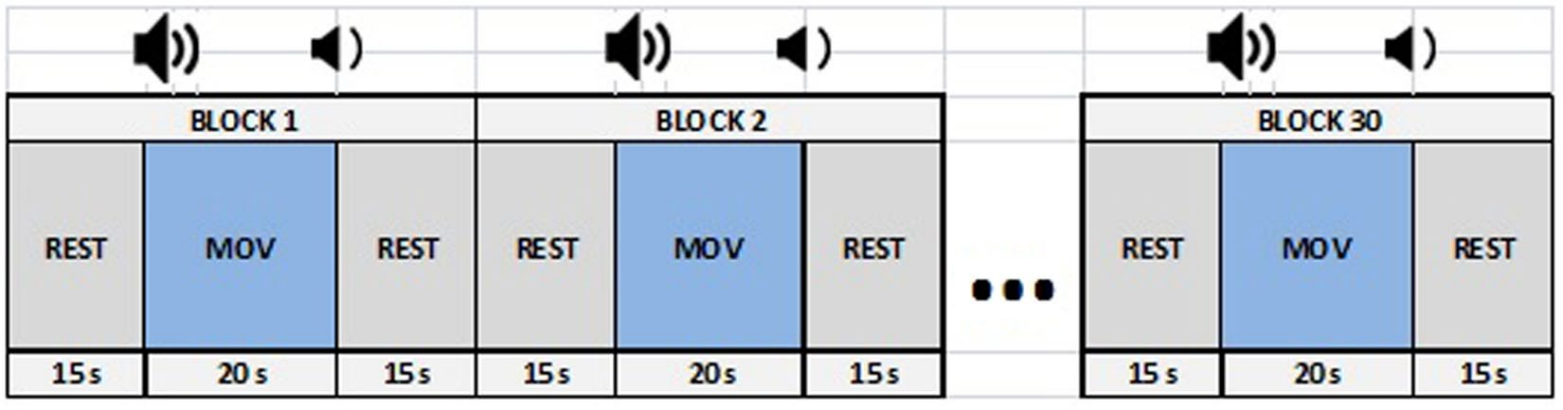
Time scheme of the protocol with indication of no movement period (REST) and movement period (MOV).

The subjects were laid down in a dark, electrically shielded room. They were instructed to start tapping a computer mouse button with their right index finger after hearing an acoustic signal. Thirty sessions of movement, each 20 s long were followed by 30 s long periods of rest. The time scheme of the experiment is shown in Fig. 4.

We applied self-paced finger movement sessions, since single movements performed after the cue did not produce a sufficiently strong fNIRS response.

### Equipment

**Figure 5:**
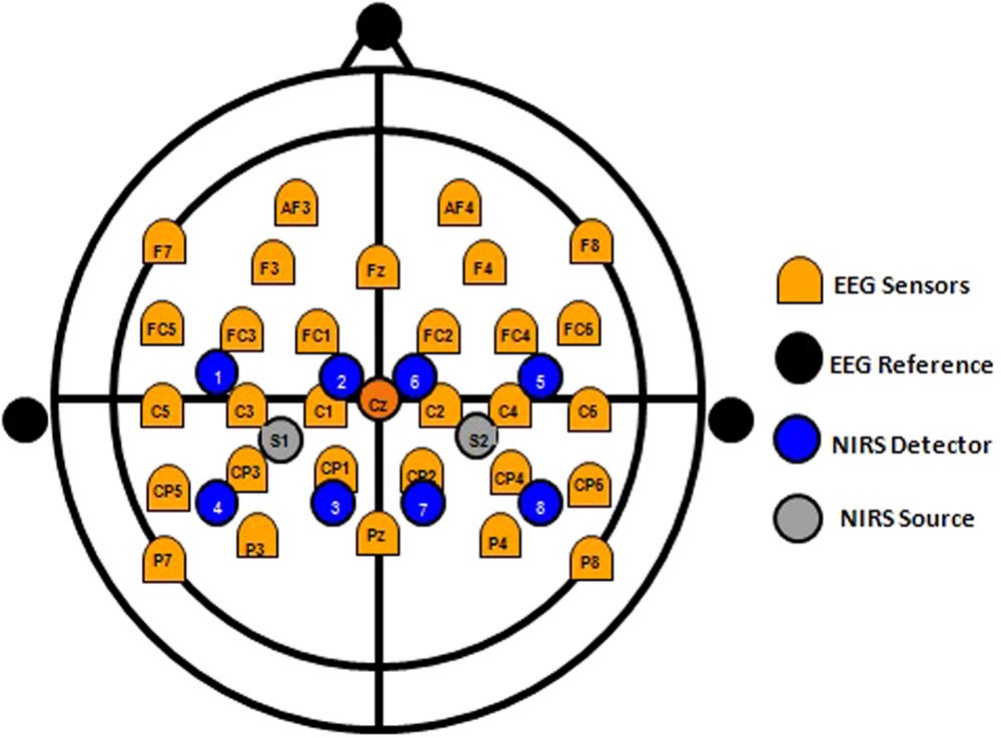
Location pattern of EEG electrodes and NIRS optodes.

EEG electrodes and fNIRS optodes were fitted in a BioSemi cap. EEG activity was recorded from 32 active Ag/AgCl electrodes (BioSemi, 10/10 system) fitted over the frontal, central and posterior head structures (Fig. 5). The electrodes impedance was kept below 1 ohm. EEG was sampled at 4096 Hz, using a 24 bits A/D converter, down-sampled to 512 Hz and filtered within the range 3–47 Hz. The reference electrodes were placed on earlobes.

A custom made time-resolved fNIRS system^29,30^ consisting of 2 optical sources and 8 detectors was placed symmetrically on both hemispheres. The emission channels were equipped with two picosecond semiconductor laser diodes operating at the wavelengths of 687 nm and 832 nm. Light pulses were generated at the frequency of 80 MHz and delivered to a healthy volunteer’s head via 2 m long optical fibers. The detection module consisted of eight photomultiplier tubes and eight time-correlated single photon counting cards. The light re-emitted from the tissue was delivered to the detection channels using eight optical fiber bundles. The tips of the fiber bundles were placed on the subject head between the EEG electrodes in such a way that their receptive field corresponded to the electrodes: C3, C1, Cp3, Cp1 in the left hemisphere and symmetrically in the right hemisphere (Fig. 5). The distance between the tips of the source fibers and detecting bundles was 3.5 cm.

### EEG processing

Hjorth transform (a small Laplace filter) was applied to the signals. The epochs with artifacts were eliminated. The data segments were synchronized with respect to the movement onset detected by the button press. For each realization for a given subject the power spectra were calculated and from the averaged spectrogram the alpha and beta rhythm bandwidths were determined. Then for the individually selected bandwidth, Event Related Desynchronisation/Synchronisation was calculated according to the formula defined by Pfurtscheller & Lopes da Silva^16^:

1 $$ERD/ERS=\frac{{P}_{m}-{P}_{r}}{{P}_{r}}\cdot 100 \% $$

where P_m_(t) means spectral power during movement and P_r_ means spectral power in the reference epoch. The movement onset was the zero point, the reference epoch was: (−15 to −2) s.

In order to construct a topographical representation, the signals were re-referenced to a common average. The ERDs in the activation period (0–20) s were normalized to z-values in order to enable a comparison between subjects. For each subject the time evolutions of ERD/ERS in the alpha and beta bands were determined.

### NIRS processing

The time-resolved NIRS system used in the study made it possible to record diffuse reflectance of short laser pulses and allowed calculation of the mean path length of photons for each subject and each source-detector pair individually. The distributions of Times of Flight of diffusely reflected photons (DTOFs) were recorded for two wavelengths in eight detection channels simultaneously. The analysis was based on the zeroth statistical moment of the DTOF – the total number of photons and the first statistical moment of the DTOF – the mean time of flight of photons <t>^31^. The mean pathlength of photons was calculated using <t> and the speed of light in the tissue of refractive index n = 1.4. Based on the mean path length values and the relative changes in the total number of the detected photons obtained at both wavelengths, the changes in concentration of HbO and HbR were determined using the Lamber-Beer equation and the molar extinction coefficients of the oxy- and deoxyhemoglobin taken from the ref.^32^.

The fNIRS signals of changes in oxy- and deoxyhemoglobine concentration were recorded at the sampling frequency of 10 Hz and the trend was removed by a linear approximation. The realizations were synchronized with respect to the movement onset and then averaged. The averaged response was smoothed by the ten points moving average window and the low pass filter of 0.4 Hz was applied.

In order to check the statistical significance the z-scores were calculated. The mean value during rest was subtracted from the mean value of the signal during movement and divided by the standard deviation during rest. Then Kolmogorow-Smirnow test (Matlab ks test) was applied. The z-scores calculated for each optode served as input values for the preparation of topographic maps which were constructed using bi-harmonic spline interpolation.

### EEG versus fNIRS

We compared the time and topographical features of fNIRS and EEG. The topographical features were illustrated by plotting the averaged over subjects ERD/ERS values for the alpha and beta bands and HbO/HbR concentrations quantified by their z-scores. The EEGLAB *topoplot* procedure involving biharmonic spline interpolation was used.

From the time evolution of the HbO, HbR and envelopes of alpha and beta activity the onsets of the neuronal and vascular responses were calculated. Those values were the times when the activation of the related signal reached 50% of its maximum, calculated after subtraction of the reference period. In order to quantify the connection between the hemodynamic and electrophysiological variables the Pearson correlationsr^2^ were calculated in the epoch −15 s to 25 s as the covariances of the two-time series divided by their standard deviations. From the correlation coefficients for individual subjects the averaged values were calculated.

### Data availability

The datasets generated during the study are available from the corresponding author on reasonable request.
